# Distal Fibular Reconstruction Using Iliac Grafting and Emslie-Vidal's Ligamentoplasty after Exeresis of Single Renal Carcinoma Metastasis

**DOI:** 10.1155/2020/8246313

**Published:** 2020-02-27

**Authors:** E. Maury, C. Granier, C. Sleth, E. Peraut, P. Maury

**Affiliations:** CHU Lapeyronie, 371 Av. du Doyen Gaston Giraud, 34090 Montpellier, France

## Abstract

*Introduction*. Restoring lateral ankle stability following distal resection of the fibula is a difficult procedure for which several surgical techniques have been proposed. Each of these techniques has potential drawbacks. This report presents a new option for fibular reconstruction. *Case Study*. We report the case of a 68-year-old male with evolving pain in the left ankle throughout the past 3 months. Three years prior to consultation, he underwent left nephrectomy for clear-cell adenocarcinoma. A swelling on the external side of the left ankle was noticed upon clinical examination, with no signs of inflammation. The ankle was stable with normal mobility. Radiographic examination revealed a 4 cm lytic lesion on the lateral malleolus with internal and external cortical damages as well as invasion of the soft tissues. Neither lower peroneotibial nor tibiotarsial joints were invaded. Needle biopsy confirmed the presence of metastatic renal clear-cell adenocarcinoma. Consequently, large exeresis of this single metastasis was indicated while preserving functional integrity of the ankle. Following block resection of the distal fibula including the lower tibioperoneal joint, a bicortical autograft was positioned to abut against the external side of the talus. Emslie-Vidal's ligamentoplasty procedure was performed with half of the short peroneal passed under the pedal flexor, then in the bone abutment, and finally through a calcaneal bone tunnel. Peroneus muscles were stabilized using a fragment sampled from the Achilles tendon. Pain decreased in 3 months, and the ankle was stable with normal functionality at a 5-year follow-up. *Discussion*. Reconstruction of the lateral ankle following fibular resection is possible by reconstructing the external facet of the malleolus using an autograft associated with Emslie-Vidal's ligamentoplasty procedure, hence stabilizing both tibiotalar and subtalar joints. This surgical procedure allowed the patient to return to his daily activities with neither instability nor evolution towards short-term tibiotalar arthrosis.

## 1. Introduction

Metastatic renal-cell carcinomas localized on the fibula are rare but occasionally require total exeresis [1, 2]. Resection of the external malleolus can induce instability of the ankle and can potentially induce arthrogenous valgus deformation [3]. Several techniques have been described for the reconstruction of the distal fibula in cases of benign or malignant tumors. The main challenge is to perform the carcinological resection while maintaining the lowest possible functional impacts on the ankle. To the best of our knowledge, Emslie-Vidal's ligamentoplasty procedure [4] associated with iliac graft in the reconstruction of the external malleolus following the exeresis of a single metastatic renal adenocarcinoma has never been described.

## 2. Case Study

**Figure 1:**
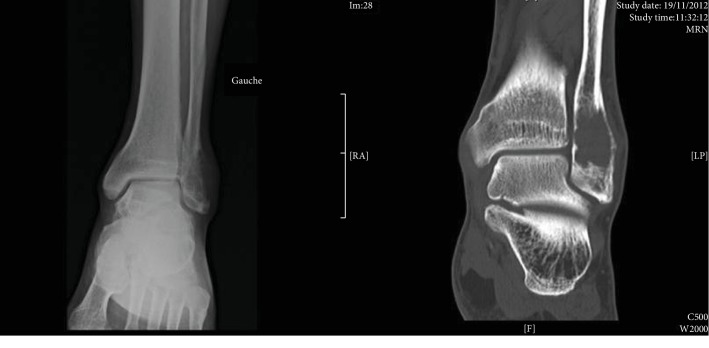
Preoperative radiological and CT-scan evaluations.

A 68-year-old male in 2012 consulted for pain in the left ankle evolving throughout the past 3 months. Three years prior to consultation, he underwent left nephrectomy for T1N0M0 clear-cell adenocarcinoma. A swelling on the external side of the left ankle was noticed upon clinical examination, with no signs of inflammation. Articular motions were not limited, with the dorsiflexion angle measured at 10° and the plantar flexion angle measured at 15°. The ankle was stable. Radiographic examination showed a 4 cm lytic lesion on the lateral malleolus. Scan images revealed osteolytic lesions along with internal and external cortical damages as well as invasion of the soft tissues (Figure 1).

Neither lower peroneotibial nor tibiotarsal joints showed signs of invasion. Scintigraphy revealed hyperfixation on the lateral malleolus. PET scan showed hypermetabolism localized on the left lateral malleolus as well as on the 7th thoracic vertebra and on the left iliac wing. MRI did not find any spinal anomalies and revealed a benign infarct on the iliac wing.

Needle biopsy confirmed the presence of a metastatic renal clear-cell adenocarcinoma. Consequently, a large exeresis of this single metastasis was required while preserving the functional integrity of the ankle.

### 2.1. Reconstruction Technique

**Figure 2:**
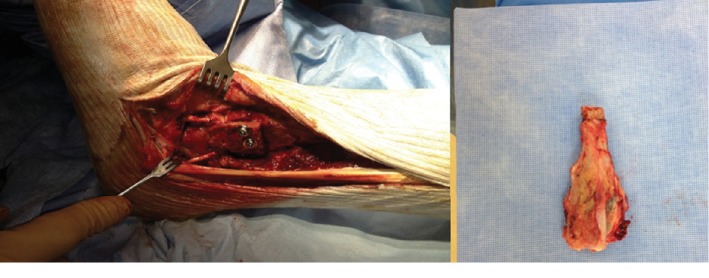
Surgical view and exeresis piece: distal fibula.

**Figure 3:**
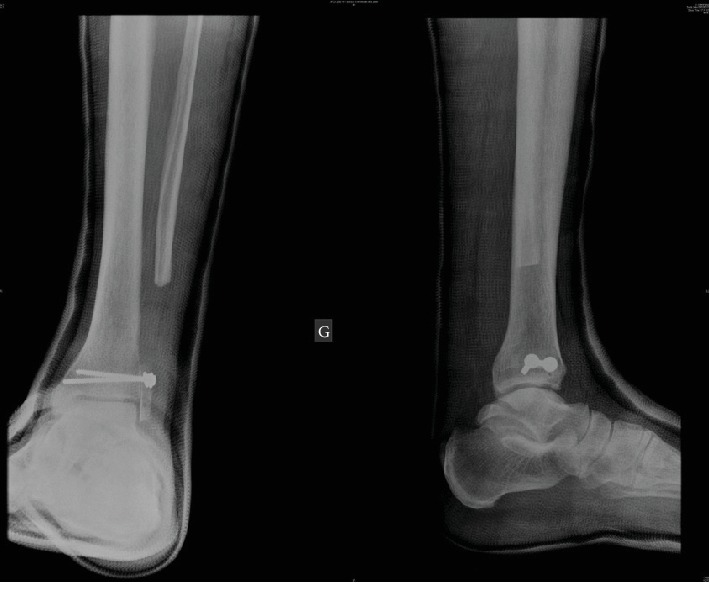
Postoperative radiography.

The patient was positioned in dorsal decubitus. The operation was carried out with a lateral approach centered on the lateral malleolus. En bloc resection of the tumor was performed, carrying the lower tibioperoneal joint. A bicortical graft was sampled from the anterior iliac crest and positioned to abut against the external side of the talus. Emslie-Vidal's ligamentoplasty procedure was performed with half of the short peroneal passed under the pedal flexor, then in the bone abutment, and finally through a calcaneal bone tunnel (Figure 2). Peroneus muscles were stabilized using a fragment sampled from the Achilles tendon. The patient was then immobilized with a cast boot for 45 days (Figure 3).

Postoperative follow-ups were simple and the patient progressively regained support over 3 months. He was helped by physiotherapy. Anatomopathology confirmed metastasis of a clear-cell renal carcinoma with nonlesional tissue edges.

#### 2.1.1. One and a Half Months Postoperative

**Figure 4:**
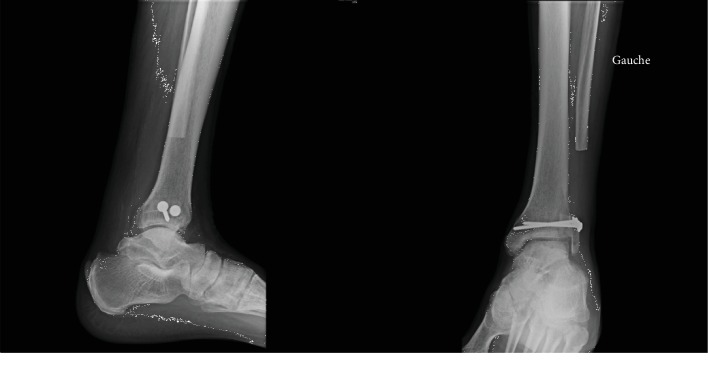
One-and-a-half-month postoperative radiography.

At one and a half months postoperative, mobility of the ankle was slightly limited (dorsiflexion +10° and plantar flexion at 15°) and control scan showed consolidation of the graft (Figure 4).

#### 2.1.2. Seven Months Postoperative

At seven months postoperative, the patient walked with a good stepping motion. The ankle was painless. Tiptoeing and walking on heels was possible. Motions were similar to the ones displayed one month postoperative with a 5° less angle for plantar flexion compared to the right ankle. The ankle was stable during clinical evaluation. Radiographic images showed integration of the graft. There was no reduction of the graft.

#### 2.1.3. One Year Postoperative

**Figure 5:**
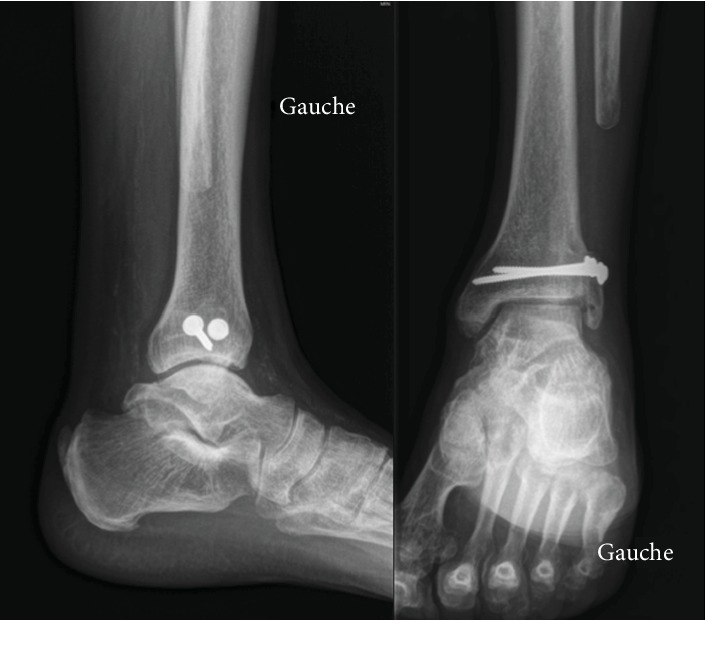
One-year postoperative radiography.

At one year postoperative, good stepping motion was found at clinical evaluation; the trophic state is good with no signs of inflammation. Ankle mobility remains unchanged with a +15° dorsiflexion during knee extension and +15° plantar flexion. There was no ankle laxity; palpation of the bone graft was slightly sensitive. There was no pain during palpation of the peroneus longus tendon or during contraction of this muscle. Palpation of the Achilles tendon was painless (Figure 5).

#### 2.1.4. 5 Years Postoperative

**Figure 6:**
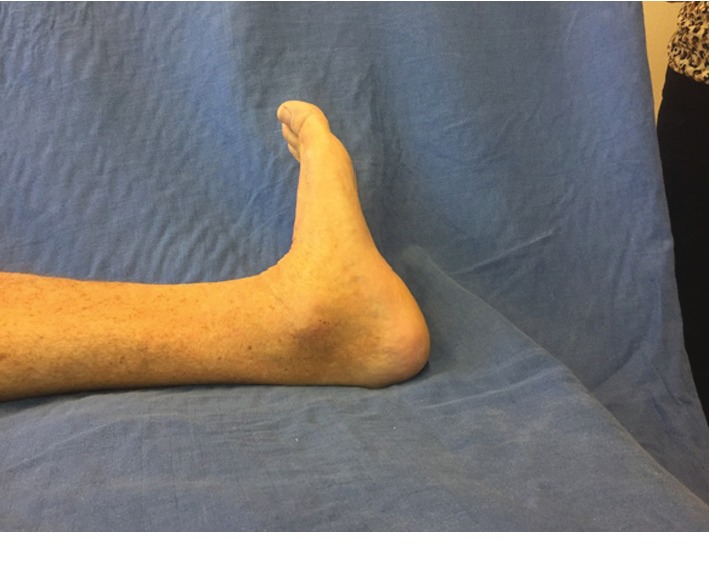
Flexion/extension image 5 years postoperative.

**Figure 7:**
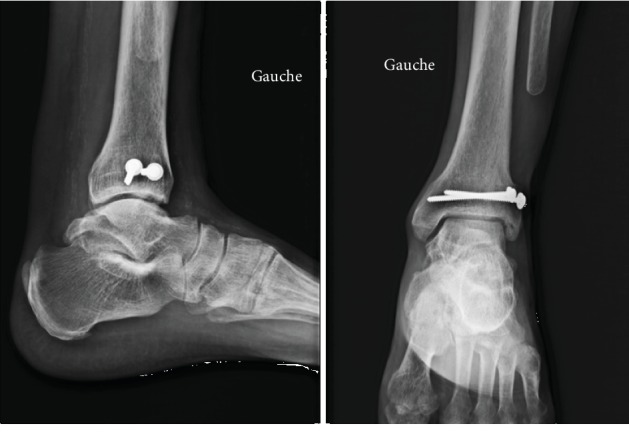
5-year postoperative radiography.

**Figure 8:**
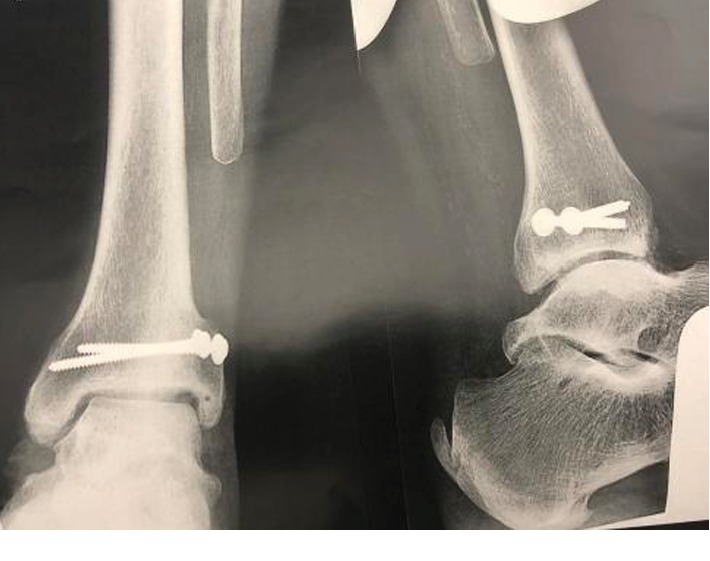
Dynamic image in forced varus and anterior drawer test 5 years postoperative.

At 5 years postoperative, there was excellent functional result (Figure 6). There was good stepping motion and a complete return to daily activities (hiking). Dorsiflexion was at 10° and 15° with extended and flexed knee, respectively. There was no instability during clinical evaluation. Radiography showed a conserved tibiotarsal interline (Figure 7) and no instability (Figure 8).

## 3. Discussion

Tumors located on the lateral malleolus are rare, including renal adenocarninoma metastases [1]. Which type of reconstruction to adopt in such case is arguable as only a few cases have been described in the literature, all using different techniques. The challenge is to restore a functional joint, while controlling for frontal laxity and valgus deformation in order to avoid evolution towards arthrosis [3, 5, 6]. Several techniques have been described for the reconstruction of the distal fibula in the case of tumoral exeresis.

Schuurman and Willems [7] described a case of reconstruction of the lateral ankle ligaments following distal fibulectomy for this type of metastasis, using a patellar tendon transplant. Postoperative motions after one year were symmetrical to the other ankle without any instability or pain.

A few cases of distal fibular reconstruction using an allograft can be found in the literature. In 1985, Lublinar et al. [8] described a case of an aneurysmal bone cyst from which functional and radiographic results were good 2.5 years postoperative.

Another case was described in 2004 by Sirveaux et al. [9] in Nancy, reporting the recurrence of chondromyxoid fibroma at the distal end of the fibula in a 25-year-old female patient. A cryopreserved allograft was set up with restoration of the lateral collateral and tibiofibular ligaments. At a two-year follow-up, the patient did not show any sign of recurrence, and the ankle was mobile, painless, and stable with comparable aesthetic features to the contralateral side.

Finally, Jamshidi et al. [10] reconstructed a distal fibula using a cryopreserved distal fibular allograft in 4 patients showing malignant tumors, resulting in complete articular motions and stability of the ankle. There was one case of postoperative infection.

Leibner et al. [11] proposed reconstruction by graft sampled from the fibula, above the resection margins of the tumoral lesion and stabilized by both syndesmotic screw and stitching of the ligaments to the graft. Results are good at one-year follow-up with indolence, stability, and conserved motions. He reviewed the literature and recommends preserving the native bone as much as possible and performing a tibiofibular syndesmosis (similar to Capanna et al. [2]) to avoid rotation of the distal fragment and the development of valgus deformation. Soft tissues including ligaments must be preserved in order to keep a stable ankle.

Capanna et al. [2] also published a series of 5 distal fibular resection cases due to malignant tumor, in two of which he performed reconstruction by graft, using the 180° rotated fibular head and fixed with a plate. In the other cases, the lateral malleolus was reconstructed and the lateral stabilization of the ankle was ensured by fibular tendon plasty.

De Gauzy et al. [12] used a vascularized ipsilateral proximal fibular graft in a 13-year-old child following osteosarcoma resection of the distal fibula, with satisfying functional and stability results. This technique presents a risk of lesion of the fibular nerve.

Ankle prosthetic reconstruction was performed by Lee et al. [13] in a 36-year-old female patient with osteosarcoma after resection of both the fibula and distal tibia with fairly good mobility (dorsiflexion 10/plantar flexion 30) and an ISOLS score of 28, suggesting good results. Nevertheless, it is an invasive procedure which should not be performed in the case of the isolated resection of the fibula.

More recently, Vaseenon et al. [14] described a transfer of the posterior tibial tendon to the fibularis brevis to restore ankle stability, which include benefits such as the absence of auto- or allograft implantation, no risk of pseudoarthrosis, no morbidity linked to the donor site, and preservation of mobility in the ankle joint. Results were satisfying at a 7-year follow-up.

Monson et al. [15] also described a new surgical technique for the stabilization of the tibiotalar joint after distal fibular resection in 3 cases with minimal perioperative morbidity for the patient, and good functional results using the fibularis brevis tendon stitched around the residual lateral ligaments of the ankle and tenodesized on the distal tibia.

Here, Emslie-Vidal's ligamentoplasty procedure associated with an iliac graft was chosen in order to reconstruct the ankle while preserving mobility and stability while allowing total exeresis of this single metastatic lesion. This procedure was the most suitable in our opinion for several reasons.

It has the advantage of using an autologous graft allowing the reconstruction of the external malleolus with a little osteosynthetic material, contrary to allograft reconstruction using osteosynthesis plates, which is nonnegligible given the cutaneous risks associated with every surgery centered on the external malleolus. The use of an autologous graft also eases the reconstruction by allowing the sampling and grafting of a piece, whose size perfectly corresponds to the morphology of the patient's talar articular surface, whereas the allograft would require a compromise on the size in cases of up- or downsizing.

Moreover, this tumoral surgery requires a rapid scheduling of the patient in order to prevent evolution of the tumor. The availability of the allograft in France is very variable and not centralized, which does not allow for carcinological surgery within short time periods [16]. Moreover, it seems problematic to us to use cryoconserved allografts in cases of malignant tumors where complementary radiography treatment or chemotherapy could be used postoperative because it would classically compromise the survival of these allografts [17].

Finally, Emslie-Vidal's ligamentoplasty associated with this autograft allows for a perfect stabilization of both tibiotalar and subtalar joints. The first part of the transplant ensures good control over stability in an equinovarus position and the second part, of which the direction is perpendicular to the subtalar plan, controls the pure varus with the ankle at a right angle at the level of the tibiotalar and subtalar joints.

Another solution would have been ankle arthrodesis with a risk of nonconsolidation and at the cost of complete limitation of motions.

At a 5-year follow-up, the 30/30 ISOLS score is representative of the very good result obtained with this technique which seemed the safest to us and the most suitable in order to obtain a satisfying functional result.

## 4. Conclusion

Block resection of the distal fibula leads to ankle dysfunctions and long-term valgus deformation as well as laxity in the frontal side with degenerative arthrosis. Our autograft technique allowed the reconstruction of this lateral malleolus along with the stabilization system of the ankle using Emslie-Vidal's ligamentoplasty with a very satisfying functional result in terms of mobility. Thanks to our procedure, this T1N0M1 patient was thus able to recover his former quality of life with a painless and functional ankle.
